# Intraosseous fluid resuscitation causes systemic fat emboli in a porcine hemorrhagic shock model

**DOI:** 10.1186/s13049-021-00986-z

**Published:** 2021-12-20

**Authors:** Steinar Kristiansen, Benjamin Storm, Dalia Dahle, Terje Domaas Josefsen, Knut Dybwik, Bent Aksel Nilsen, Erik Waage-Nielsen

**Affiliations:** 1 Surgery and Intensive Care Unit, University Hospital of Northern Norway, Tromsø, Norway; 2 Department of Surgery, Nordland Hospital Trust, Bodø, Norway; 3 Department of Clinical Medicine, Faculty of Health Sciences, Arctic University of Norway, Tromsø, Norway; 4 Research Laboratory, Nordland Hospital Trust, Bodø, Norway; 5 Faculty of Biosciences and Aquaculture, Nord University, Bodø, Norway; 6 Faculty of Nursing and Health Sciences, Nord University, Bodø, Norway; 7 Department of Immunology, Oslo University Hospital, University of Oslo, Oslo, Norway

**Keywords:** Fat embolism, Fat embolism syndrome, Systemic embolization, Intraosseous cannulation, Coronary fat embolism, Open chest conditions

## Abstract

**Background:**

Intraosseous cannulation can be life-saving when intravenous access cannot be readily achieved. However, it has been shown that the procedure may cause fat emboli to the lungs and brain. Fat embolization may cause serious respiratory failure and fat embolism syndrome. We investigated whether intraosseous fluid resuscitation in pigs in hemorrhagic shock caused pulmonary or systemic embolization to the heart, brain, or kidneys and if this was enhanced by open chest conditions.

**Methods:**

We induced hemorrhagic shock in anesthetized pigs followed by fluid-resuscitation through bilaterally placed tibial (hind leg) intraosseous cannulas. The fluid-resuscitation was limited to intraosseous or i.v. fluid therapy, and did not involve cardiopulmonary resuscitation or other interventions. A subgroup underwent median sternotomy with pericardiectomy and pleurotomy before hemorrhagic shock was induced. We used invasive hemodynamic and respiratory monitoring including Swan Ganz pulmonary artery catheter and transesophageal echocardiography and obtained biopsies from the lungs, heart, brain, and left kidney postmortem.

**Results:**

All pigs exposed to intraosseous infusion had pulmonary fat emboli in postmortem biopsies. Additionally, seven of twenty-one pigs had coronary fat emboli. None of the pigs with open chest had fat emboli in postmortem lung, heart, or kidney biopsies. During intraosseous fluid-resuscitation, three pigs developed significant ST-elevations on ECG; all of these animals had coronary fat emboli on postmortem biopsies.

**Conclusions:**

Systemic fat embolism occurred in the form of coronary fat emboli in a third of the animals who underwent intraosseous fluid resuscitation. Open chest conditions did not increase the incidence of systemic fat embolization.

**Supplementary Information:**

The online version contains supplementary material available at 10.1186/s13049-021-00986-z.

## Background

The establishment of intraosseous access can be a life-saving procedure in critically ill patients when venous access cannot be readily achieved by other means [1, 2]. In critically ill patients, intraosseous access is faster than central venous access [3], and faster [4], or as fast as [5], peripheral intravenous access. Although the procedure is considered to be safe, reported complication rates vary considerably from under 1% [1], to over 12% [2, 6].

Intraosseous administration of fluids causes fat embolization to the lungs in both clinical case studies and animal studies [3, 7–13]. Systemic fat embolization, however, has only been seen after trauma or orthopedic surgery [14–16], and it may occur in the absence of an intracardiac shunt [17–20]. To our knowledge, only one case report has described systemic fat embolization after intraosseous cannulation [13], and the phenomenon has not been previously studied in animals.

It has been proposed that open chest, e.g. after emergency thoracotomy [21], may increase the occurrence of systemic air embolization [22]. We, therefore, hypothesized that intraosseous fluid resuscitation would generate both pulmonary and systemic fat emboli, and that open chest would increase systemic embolization.

Thus, our study aimed to examine whether intraosseous fluid resuscitation caused systemic fat embolization, and how this was influenced by open chest conditions. The fat emboli were sought for in postmortem biopsies of the lungs, heart, brain, and left kidney (Table 1).

**Table 1:** Postmortem histopathological examination for fat in selected organs

Organ	Open chest (n = 7)	Closed chest (n = 14)	Sham (n = 2)	Control (n = 3)
Lung	7 (100%)	14 (100%)	0 (0%)	3 (100%)
Heart	0 (0%)	7 (50%)	0 (0%)	0 (0%)
Brain	0 (0%)	0 (0%)	0 (0%)	0 (0%)
Kidney	0 (0%)	0 (0%)	0 (0%)	0 (0%)

## Methods

### Aim, design, and setting

**Fig. 1:**
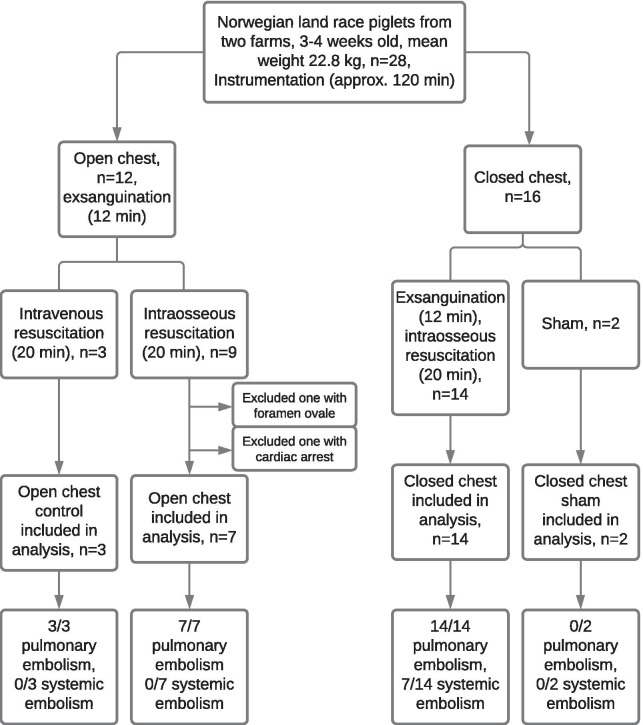
Inclusion of animals. We allocated 28 pigs to receive intraosseous infusion after induced hemorrhagic shock with either open or closed chest or to serve as sham animals. Open chest animals underwent midline sternotomy

In this two-center, non-randomized experimental study, we studied whether intraosseous infusion therapy causes systemic fat embolization in a porcine model for hemorrhagic shock and intraosseous fluid resuscitation. The study design is summarized in Fig. 1.

### Experimental animals

The Norwegian Animal Research Authority approved the study (FOTS ID 19803), and we performed the experiments under the Norwegian Laboratory Animal Regulations and the EU directive 2010/63/EU.

We allocated Norwegian landrace pigs from two farms to undergo intraosseous crystalloid fluid resuscitation following induced hemorrhagic shock, either with midline sternotomy, open pleura, and pericardium (open chest) or closed chest.

Additionally, two animals served as sham, undergoing only standard instrumentation and anesthesia. One animal with patent foramen ovale and one animal with perioperative cardiac arrest were excluded.

### Instrumentation, anesthesia, and monitoring

Anesthesia, instrumentation, and euthanasia were as previously described by Storm et al. [22], elaborated in Additional file 1. Additionally, a Secalon-T Emergency Catheter MeritMedical, USA) was placed in the left femoral artery.

### Exsanguination, intraosseous infusion, and data collection

The intraosseous cannula was inserted in the tibial tuberosity bilaterally (hind legs) using the Arrow EZ-IO intraosseous vascular access system (Teleflex, USA). Cannulation of the marrow cavity was confirmed by loss of resistance on the needle upon entry into the marrow cavity, gentle aspiration of bone marrow, free fluid flow into the bone by injection of 10 ml saline, and detectable fluid bolus in the right ventricular outlet tract by transesophageal echocardiography. After induction and instrumentation, the pigs were exsanguinated through the catheter in the left femoral artery at a rate of 50 ml/min, until a mean arterial pressure (MAP) of 30 mmHg was reached and an arterial blood sample was obtained. After exsanguination, all pigs were resuscitated with an infusion of Lactated Ringers Solution pressurized to 300 mmHg at a rate of approximately 100 ml/min through both intraosseous cannulas until the MAP was 65 mmHg. Infusions were maintained by repeated use of 20 ml syringes connected to pressurized Lactated Ringers Solution. Sham and control animals did not receive intraosseous Lactated Ringers infusion. This method of resuscitation was chosen because it is applied in some emergency clinical settings.

Pigs allocated to the control group underwent sternotomy and were exsanguinated as described above, but fluid resuscitation was administered through intravenous catheters inserted bilaterally in large ear veins.

Pigs allocated to the sham group were not exsanguinated or fluid resuscitated. We euthanized the animals 300 min after the start of the intraosseous infusion by central venous injection of potassium chloride.

**Fig. 2:**
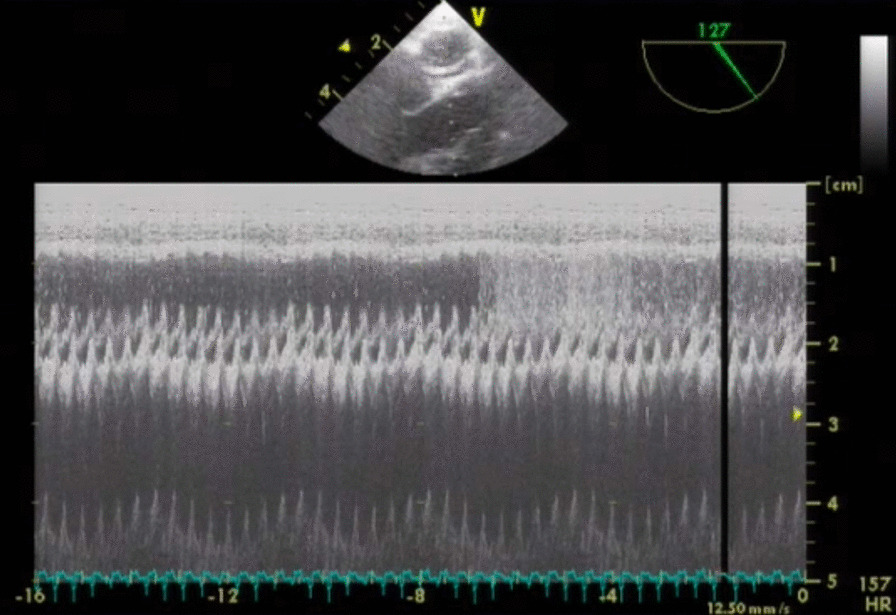
Transesophageal M-mode echocardiography through the pulmonary artery and the left ventricular outlet tract. Transesophageal M-mode echocardiography (TEE) visualizing the pulmonary artery (above) and the left ventricular outlet tract (below) during intraosseous fluid infusion. We used continuous TEE to help confirm intraosseous placement and to monitor for intracardiac shunt. Horizontal sweep rate was set to 12.50 mm/s

We used continuous M-mode and intermittent 2D transesophageal echocardiography (TEE) to verify intraosseous infusion and to detect the systemic passage of emboli by obtaining an echocardiographic window of both the pulmonary artery and the left ventricular outlet tract (LVOT) as described by Storm et al. [22]. We registered elapsed time and eventual systemic passage of emboli (Fig. 2).

Postmortem, we examined the heart, lungs, left kidney, cerebrum, cerebellum, and the medulla oblongata and obtained biopsies. We screened for intracardiac shunt by echocardiography and postmortem we examined the heart for septal defects such as patent foramen ovale.

### Histopathological analyses

Tissue samples of brain, lung, heart and kidney size 1 × 1 × 0.3 cm were frozen in the OCT compound on dry ice. Sections 8 µm thick were cut at − 20 °C by use of a cryostat (Cryostar NX50, Thermo Scientific, USA). Three to five serial sections were mounted on poly-L-lysine coated slides and air-dried before staining. The sections were fixed with 4% neutral buffered formalin for 20 min., rinsed in tap water, following by few dips in 60% isopropanol and incubation in 0.5% Oil Red O working solution (30 mL 0.5% Oil Red O (Sigma-Aldrich, St Louis, MO) stock solution diluted with 20 mL 1% dextrin aqueous solution) for 20 min. After incubation samples were shortly rinsed in 60% isopropanol and counterstained with Gill III modified Hematoxylin solution (Merck KGaA, Darmstadt, Germany) for 15 s, rinsed 3 × 30 s with distilled water, blued, and mounted with glycerin jelly. Oil Red O stained images were captured by using Olympus SC180 digital camera (Olympus Europa GmbH, Hamburg, Germany) installed on Olympus BX51 light microscope.

The images were processed by using Olympus cellSens Entry software Soft Imaging System GmbH, Munster, Germany). The staining methods are described in Bancroft’s Theory and Practice of Histological Techniques [23].

### Power calculation

We expected to find systemic fat emboli in 80% of the animals with an open chest, and 10% of the animals with a closed chest, based on previous experience with similar animal models using intravenously injected air [22]. An online sample calculator (https://clincalc.com/stats/samplesize.aspx) with a 2:1 enrolment ratio, alpha of 5%, and power of 80%, yielded a group-size of 14 with 7 pigs in each group allocated to either open or closed chest.

### Statistics

We used Prism 9 for Mac (Graphpad Software, San Diego, California USA) for statistical calculations and used restricted maximum likelihood mixed model (REML) analysis where statistical comparison was relevant, after testing for normality. We considered a *p* < 0.05 significant.

## Results

### Detection of systemic and pulmonary fat emboli

By continuous TEE we observed hyperechogenic material consistent with ongoing fluid infusion in the pulmonary artery following intraosseous flush and infusion. Infusion fluid or emboli were not detected in the LVOT, except in the one animal with patent foramen ovale which was excluded.

We found widespread pulmonary fat emboli in all pigs except sham animals in postmortem biopsies. In seven of 21 pigs in the intraosseous group, we found fat emboli in coronary arteries of the left ventricle. All pigs with coronary fat emboli were from the closed chest group. We did not find fat emboli in renal biopsies or biopsies from the dorsal and ventral cerebrum and cerebellum.

### Clinical manifestations of fat embolization

#### Pulmonary manifestations

Only pigs with open chest developed a mild oxygenation failure with PaO2:FiO2 ratio at 301 mmHg, compared to pigs with closed chest, which had normal oxygenation, with an average PaO2:FiO2 ratio of 363 (*p* = 0.04).

#### Cardiovascular manifestations

5-lead ECG revealed significant ST-elevation (> 1 mm ST-elevation in minimum two standard leads lasting > 15 min) following intraosseous resuscitation in three animals in the closed group, who all were found to have cardiac fat embolization post-mortem. There were no further signs of cardiovascular deterioration.

Post-mortem macroscopic examination of the heart and lungs did not reveal marked pathology.

#### Time to death after intraosseous infusion

All pigs survived throughout the 300 min experiments without interventions other than maintenance fluid therapy.

## Discussion

In a porcine model, we have shown that intraosseous fluid resuscitation causes systemic fat embolization in addition to pulmonary emboli. Our findings suggest that intraosseous cannulation and/or infusion may cause widespread pulmonary fat emboli, supporting current literature [8, 9].

Three control animals also developed pulmonary fat emboli, which we believe came from the median sternotomy, a procedure known to cause fat embolization [24–26].

Intraosseous fluid resuscitation caused fat emboli to be dislodged in the coronary vessels of the left ventricle in animals with closed chests. To our knowledge, systemic fat embolization confirmed by biopsies following intraosseous cannulation and/or infusion has not been described in an animal model. Systemic fat embolization following intraosseous infusion has been documented in one single clinical case [13]. We found cardiac fat emboli only in pigs with closed chest. This may suggest, with the limitation of few pigs in the series, that an open chest does not reduce the threshold for when fat reaches the systemic circulation through the lungs, as is the case concerning air emboli [22].

The pigs did not develop clinical fat embolism syndrome for the duration of the experiments, a finding consistent with previous literature [9]. We observed significant ST-elevations in three animals who were found to have coronary fat emboli. The lack of major clinical deterioration may be explained by the fact that fat embolism syndrome usually occurs 24–72 h after the embolic event [16, 27–29].

**Fig. 3:**
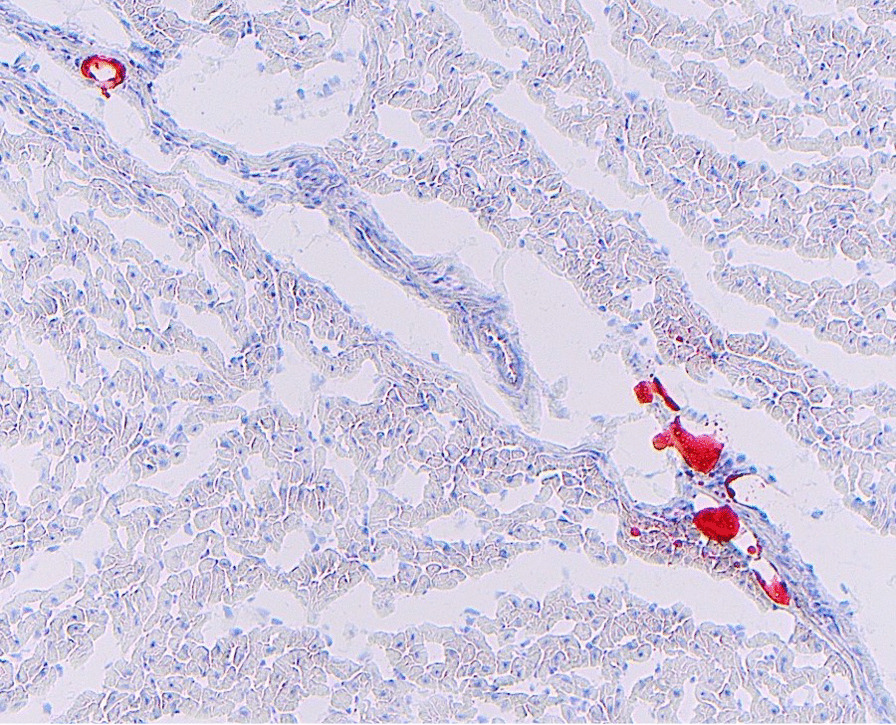
Frozen myocardial tissue stained Oil RedO and counterstained with Hematoxylin Gill III. Left ventricle of the heart, Oilred O stain on frozen tissue conterstained with Hematoxylin Gill III showing several intravascular fat emboli (100× total magnification)

We analyzed several anatomical separate slices of each animal’s brain and kidneys but did not find convincing evidence of intravascular fat emboli. The fact that systemic fat emboli were only found in coronary vessels may be random, and the lack of emboli in the brain and renal biopsies does not exclude the possibility that embolization to these tissues occurred. Moreover, preparation of brain tissue for oilred O staining proved challenging, which may have caused us to miss cerebral fat emboli. More numerous biopsies and improved technique for preparing brain tissue would increase the sensitivity in discovering further systemic embolization. Cerebral magnetic resonance imaging (MRI) with susceptibility weighted imaging (SWI) would to a high degree of certainty reveal fat embolization to the brain, as has been shown clinically [15, 30–32] (Fig. 3).

**Fig. 4:**
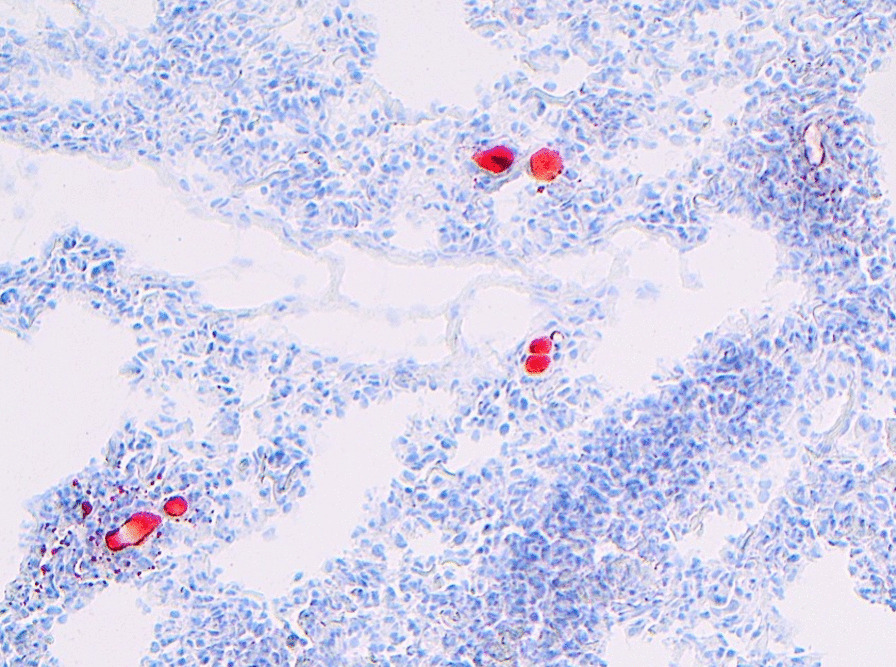
Frozen pulmonary tissue stained Oil RedO and counterstained with Hematoxylin Gill III. Posterior lobe of left lung, Oilred O staining counterstained with Hematoxylin Gill III on frozen tissue showing multiple intravascular fat emboli (100× total magnification)

Diagnosing fat embolism syndrome in patients can also prove difficult. Biopsies are only obtained from the minority of patients who die, and if they are obtained, biopsy material is not routinely stained with oilred O to identify fat emboli. The more common symptoms from fat embolism syndrome such as respiratory failure and neurological symptoms have low sensitivity, with the typical petechial rash having higher spesifisity but occurring in only 20–50% of patients [33]. As cerebral fat emboli cause petechial hemorrhages, magnetic resonance imaging (MRI) with susceptibility weighted imaging (SWI) is perhaps the most valuable diagnostic tool in living patients [13, 32, 34]. However, our experience is that magnetic resonance imaging with SWI weighted imaging is not routinely performed, and further, that MRI is obtained only rarely if fat embolism syndrome is suspected (Fig. 4).

### Systemic embolization

Systemic passage of emboli occurred in several animals, in all cases in the form of coronary embolization, and only in animals with closed chest. Systemic passage – or shunting – occurred in the closed chest group as evident by the presence of coronary fat emboli. None of the included animals had an intracardiac shunt. Systemic passage of emboli in the absence of intracardiac shunt has been shown in humans and animals [20, 35–38]. Venous emboli can theoretically enter the systemic circulation through at least three sites; the pulmonary capillary network, an intracardiac shunt – for example an open foramen ovale—or through extra-alveolar shunt-vessels, more commonly referred to as anatomical shunts [35, 39]. It has been proposed that intravascular fat emboli may be “squeezed through” the pulmonary capillary network, i.e. that small pulmonary fat emboli may migrate through the pulmonary capillary network and cause systemic embolization [20].

In addition, extra-alveolar shunts exist in healthy humans [35, 39, 40], likely contributing to systemic embolization. The extent of shunting through these vessels is probably dynamic, as the shunting appears to increase with hypoxia, posture change, and exercise [35, 39, 40].

In our experiments, we hypothesized that an open chest would increase systemic fat embolization. We believed that an open chest would cause reduced transmural lung pressure, allowing over-expansion of the lungs. This, in conjunction with mechanical ventilation, has been proposed to facilitate the recruitment of extra-alveolar shunt vessels, thus opening up dynamic passages available for systemic shunting for air emboli [22, 41]. Fat emboli likely do not share the gravitational, thrombogenic, or physical properties of air emboli, and are probably influenced by the mentioned factors in other ways.

### Clinical relevance

We have demonstrated that intraosseous infusion causes pulmonary and potentially coronary fat embolization, and that systemic embolization did not occur in animals with open chest. Intraosseous infusion is used when intravenous access is difficult, as is often the case in patients with serious hypovolemia or hypotension. In critically ill patients, delayed fluid or drug administration may be fatal. Intraosseous access may be warranted in time-critical situations, despite a high probability of pulmonary emboli and a risk of coronary and cerebral embolization, as these emboli may commonly be subclinical. Thus, open chest situations, as with thoracic trauma and emergency thoracotomy, do not by itself seem to contraindicate intraosseous cannulation.

## Limitations

Our study has potential limitations. A subgroup of pigs was exposed to both sternotomy and intraosseous infusion, and both interventions are known to cause fat embolization, possibly placing these pigs at a higher risk of systemic passage of fat emboli. Despite this, no coronary fat emboli were observed in pigs with open chest. Thus, it is reasonable to conclude that the intraosseous infusion, and not the sternotomy, was the most significant source of fat emboli in both pigs with open and closed chest. We conducted the experiments at two laboratories, possibly introducing a bias in our findings. However, as all experiments were conducted by the same researchers using identical protocols, medications, and equipment is is not likely that this has affected our findings to any extent. Further, the animals were of the same race and bred from the same national insemination stock and supplier. Echocardiography was performed by two different researchers, and neither detected passage of systemic fat emboli, even in cases where fat emboli were found in postmortem left ventricle biopsies, suggesting that the TEE had a low sensitivity for the detection of the systemic passage of fat emboli.

The distribution of systemic fat emboli in the tissues is unpredictable, and despite acquiring several tissue biopsies postmortem, we sampled only a fraction of the organs, and organ embolization may indeed have occurred without us detecting them in the sampled tissue. Finally, it is established that clinical fat embolism syndrome usually does not manifest itself until 24–48 h after fat embolization has occurred [16, 29, 42]; our observational period was limited to 300 min. Thus, we cannot exclude the possibility that the fat emboli would later have caused fat embolism syndrome.

## Conclusions

Pulmonary fat emboli were ubiquitous during intraosseous infusion and systemic passage of fat emboli frequently occurred, but did not cause major respiratory or cardiovascular deterioration. Open chest conditions did not increase the risk of systemic passage of fat emboli.

## Supplementary Information

**Additional file 1.** Methods and materials.

## Data Availability

The datasets from the experiments are available from the corresponding author on request.
