# Supplementary material for: Intraosseous fluid resuscitation causes systemic fat emboli in a porcine hemorrhagic shock model
Source: Scand J Trauma Resusc Emerg Med. 2021 Dec 20;29:172. doi: 10.1186/s13049-021-00986-z (PMC8686379; doi:10.1186/s13049-021-00986-z)
Supplement: Supplementary file 1 — Additional file 1. Methods and materials. [file 13049_2021_986_MOESM1_ESM.docx]

**Appendix 2 – Methods**

**Aim, design, and setting**

In this two-center, non-randomized experimental study, we studied whether intraosseous infusion therapy causes systemic fat embolization in a porcine model for hemorrhagic shock and intraosseous fluid resuscitation. The study design is summarized in Figure 1.

**Experimental animals**

The Norwegian Animal Research Authority approved the study (FOTS ID 19803), and we performed the experiments under the Norwegian Laboratory Animal Regulations and the EU directive 2010/63/EU.

We allocated Norwegian landrace pigs from two farms to undergo intraosseous crystalloid fluid resuscitation following induced hemorrhagic shock, either with midline sternotomy, open pleura, and pericardium (open chest) or closed chest.

Additionally, two animals served as sham, undergoing only standard instrumentation and anesthesia. One animal with patent foramen ovale and one animal with perioperative cardiac arrest were excluded.

**Instrumentation, anesthesia, and monitoring**

We anesthetized all pigs with azaperone 40 mg, ketamine 500 mg, and atropine 0.5 mg intramuscularly and maintained the anesthesia with an intravenous infusion of morphine 2 mg/kg/h, midazolam 0.15 mg/kg/h and pentobarbital 4 mg/kg/h. We endotracheally intubated the pigs with a 6 mm outer diameter tube. We mechanically ventilated the pigs with a tidal volume of 10-15 mL/kg, a rate of 20/min, and zero positive end-expiratory pressure. Tidal volume and respiratory rate were adjusted to maintain a pH of 7.35-7.45. Inspiratory oxygen fraction (FiO_2_) was adjusted to maintain an arterial pulse oximetry saturation (SpO_2_) above 93%. We infused Ringer's acetate to compensate for insensible fluid losses with a rate of 2-3 mL/kg/h in pigs with closed thorax and 10 mL/kg/h in pigs with open chest and pericardium. In situations where MAP dropped below 55 mm Hg after intraosseous fluid resuscitation, the pigs were resuscitated with repeated boluses of 100 mL Ringer's acetate and infusion of noradrenaline in the range 0.01 to 0.8 μg/kg/min.

We surgically inserted an arterial line in the internal carotid artery, a 7.5 Fr pulmonary artery catheter (Edwards Vigilance Swan-Ganz CCOmbo) through the internal jugular vein, a central venous catheter (B. Braun CVC Certofix) through the external jugular vein, a 4 Fr 8 cm PICCO thermodilution catheter (Pulsion/Getinge) in the femoral artery, a Secalon-T Emergency Catheter (MeritMedical, USA) in the left femoral artery and a suprapubic catheter with a temperature sensor in the bladder. We placed a pediatric 9T transesophageal echo-probe (General Electric) in an upper-esophageal position and connected it to either a Vivid 7 pro (General Electric) or a Vivid E9 (General Electric) echo machine. We recorded ECG, ST-segment, SpO_2_, expired end-tidal CO_2,_ and continuous invasive arterial-, pulmonary-, and central-venous pressures on either a Solar monitor (General Electric) or SC8000 monitor (Siemens Healthcare). We drew arterial blood gasses approximately hourly throughout the experiments and immediately before death and analyzed these on the ABL Flex 80 (Radiometer).

Pigs allocated to open chest and pericardium had a sternotomy with an opening of the pleural sac and pericardium. We attached M-mode sensors (Imasonic SAS) onto the left and right ventricle, and a combined six-axis gyroscope and accelerometer MPU6050 (Invensense Inc) onto the right ventricle of the heart (Supporting Information **1A**). We retrieved recordings from the M-mode sensors using a custom-built computer and software (The Intervention Centre, Oslo University Hospital).

Before the start of the experiment, we performed a transesophageal echocardiogram (TEE) in all pigs, including an agitated saline test to examine for patent foramen ovale, and we then positioned the transesophageal echo probe to visualize the aorta and the right ventricular outlet tract (RVOT) or the pulmonary artery or the left atrium and the RVOT. We repeatedly assessed right and left ventricular dimension and function throughout the experiments.

We recorded cardiac output by hourly thermodilution (average of three injections of 5 mL ice-cold Ringer's Acetate) by the Edwards Vigilance II (Edwards) and Pulsion PICCO2 (Pulsion/Getinge).

**Exsanguination, intraosseous and intravenous infusion, and data collection**

The intraosseous cannula was inserted in the tibial tuberosity bilaterally (hind legs) using the Arrow EZ-IO intraosseous vascular access system (Teleflex, USA). Cannulation of the marrow cavity was confirmed by loss of resistance on the needle upon entry into the marrow cavity, gentle aspiration of bone marrow, free fluid flow into the bone by injection of 10 ml saline, and detectable fluid bolus in the right ventricular outlet tract by transesophageal echocardiography. After induction and instrumentation, the pigs were exsanguinated through the catheter in the left femoral artery at a rate of 50 ml/minute, until a mean arterial pressure (MAP) of 30 mmHg was reached and an arterial blood sample was obtained. After exsanguination, all pigs were resuscitated with an infusion of Lactated Ringers Solution pressurized to 300 mmHg at a rate of approximately 100 ml/minute through both intraosseous cannulas until the MAP was 65 mmHg. Infusions were maintained by repeated use of 20 ml syringes connected to pressurized Lactated Ringers Solution. Sham and control animals did not receive intraosseous Lactated Ringers infusion. This method of resuscitation was chosen because it is applied in some emergency clinical settings.

Pigs allocated to the control group underwent sternotomy and were exsanguinated as described above, but fluid resuscitation was administered through intravenous catheters inserted bilaterally in large ear veins.

Pigs allocated to the sham group were not exsanguinated or fluid resuscitated. We euthanized the animals 300 minutes after the start of the intraosseous infusion by central venous injection of potassium chloride.

We used continuous M-mode and intermittent 2D transesophageal echocardiography (TEE) to verify intraosseous infusion and to detect the systemic passage of emboli by obtaining an echocardiographic window of both the pulmonary artery and the left ventricular outlet tract (LVOT) as described by Storm et al [24]. We registered elapsed time and eventual systemic passage of emboli.

Postmortem, we examined the heart, lungs, left kidney, cerebrum, cerebellum, and the medulla oblongata and obtained biopsies. We screened for intracardiac shunt by echocardiography and postmortem we examined the heart for septal defects such as patent foramen ovale.

**Histopathological analyses**

Tissue samples of brain, lung, heart, and kidney size 1x1x0.3 cm were frozen in the OCT compound on dry ice. Sections 8µm thick were cut at -20 °C by use of a cryostat (Cryostar NX50, Thermo Scientific, USA). Three to five serial sections were mounted on poly-L-lysine coated slides and air-dried before staining. The sections were fixed with 4% neutral buffered formalin for 20 min., rinsed in tap water, following by few dips in 60% isopropanol and incubation in 0.5% Oil Red O working solution (30 mL 0.5% Oil Red O (Sigma-Aldrich, St Louis, MO) stock solution diluted with 20 mL 1% dextrin aqueous solution) for 20 min. After incubation samples were shortly rinsed in 60% isopropanol and counterstained with Gill III modified Hematoxylin solution (Merck KGaA, Darmstadt, Germany) for 15 sec, rinsed 3x30 sec with distilled water, blued, and mounted with glycerin jelly. Oil Red O stained images were captured by using Olympus SC180 digital camera (Olympus Europa GmbH, Hamburg, Germany) installed on Olympus BX51 light microscope.

The images were processed by using Olympus cellSens Entry software Soft Imaging System GmbH, Munster, Germany). The staining methods are described in Bancroft’s Theory and Practice of Histological Techniques [25].

**Power calculation**

We expected to find systemic fat emboli in 80% of the animals with an open chest, and 10% of the animals with a closed chest, based on previous experience with similar animal models using intravenously injected air [24]. An online sample calculator (https://clincalc.com/stats/samplesize.aspx) with a 2:1 enrolment ratio, alpha of 5%, and power of 80%, yielded a group-size of 14 with 7 pigs in each group allocated to either open or closed chest.

**Statistics**

We used Prism 9 for Mac (Graphpad Software, San Diego, California USA) for statistical calculations and used restricted maximum likelihood mixed model (REML) analysis where statistical comparison was relevant, after testing for normality. We considered a p<0.05 significant.
